# Exploring the adaptive leisure activities of classified nursing model in elderly colon cancer patients: a perspective on interactive care

**DOI:** 10.1186/s12904-023-01317-y

**Published:** 2023-12-12

**Authors:** Yun-Zhao Lin, Po Chen, Meng-Meng Lin, Jia-Li Chen, Min-Min Shi, Feng Guo

**Affiliations:** 1 Hangzhou City University, Hangzhou, China; 2 Saint Louis University, Baguio, Philippines; 3 Zhejiang Wanli University, Ningbo, China; 4 Ningbo Wanli Counterpart Cooperation and Anti-Poverty Research Institute, Ningbo, China; 5 Cangnan County People’s Hospital, Wenzhou, China; 6 School of Nursing, Zhejiang Chinese Medical University, Hangzhou, China; 7 School of Management, Wenzhou Business College, Wenzhou, China

**Keywords:** Leisure nursing, Adoptive activities, Colon cancer, Aged, Emotional traits, Self-care behavior

## Abstract

**Objective:**

The aims of the study were first to explore the adaptive leisure activities of classified nursing model from the perspective of nurse-patient interactive care, and to explore its impact on the physical and mental health of patients with colon cancer.

**Methods:**

From September 2017 to March 2022 as the observation time node, 82 patients with colon cancer who met the established inclusion and exclusion criteria were regarded as the research objects through the random number table as the grouping tool. The two groups of patients were named as the research group and the control group, with 41 patients in each group. The control group implemented routine nursing measures, and the research group implemented classified nursing mode and adaptive leisure activity mode. The two groups of patients received 4 weeks of nursing intervention. With the help of self-rating anxiety scale, self-rating depression scale, self-care ability evaluation scale and health status survey brief form, the two groups of patients were compared before intervention and at the end of the 4th week after intervention.

**Results:**

After the intervention, the anxiety score (*t* = 6.656, *p* < 0.001) and depression score (*t* = 4.851, *p* < 0.001) of the research group were lower than those of the control group, and the difference was statistically significant. After the intervention, the self-concept (*t* = 4.845, *p* < 0.001), self-responsibility (*t* = 6.071, *p* < 0.001), self-care skills (*t* = 3.341, *p* < 0.001), health knowledge (*t* = 3.698, *p* < 0.001) and total score (*t* = 9.246, *p* < 0.001) of the research group were higher than those of the control group, and the difference was statistically significant. After the intervention, physical functioning (*t* = 8.141, *p* < 0.001), bodily pain (*t* = 6.083, *p* < 0.001), general health (*t* = 9.424, *p* < 0.001), role-physical (*t* = 8.057, *p* < 0.001), role-emotional (*t* = 13.252, *p* < 0.001), mental health (*t* = 12.565, *p* < 0.001), social functioning (*t* = 10.813, *p* < 0.001) and vitality score (*t* = 12.890, *p* < 0.001) of the research group were higher than those of the control group, with significant differences.

**Conclusion:**

Interactive care through adaptive leisure nursing improves mental well-being, self-management, and psychosocial functioning in elderly colon cancer patients, promoting overall health.

## Introduction

Colon cancer is a common digestive system tumor in clinic. The physical pain and psychological distress caused by this disease will have a great impact on the quality of life of patients [1]. Colon cancer is more common in the age group of 65–74, and the incidence of colon cancer is showing an increasing trend [2]. Most of the patients are elderly and therefore had a stronger negative perception of the disease [3]. In addition, the superposition of comprehensive treatment methods such as radiotherapy, chemotherapy and surgery will further increase the individual’s negative emotional perception at the spiritual and emotional level, indirectly affect the individual’s social function, weaken the basic self-care level, and even induce obvious physical and mental stress events [4]. Therefore, a multivariate analysis was performed on the personality traits, intrinsic needs, behavioral attitudes and cognitive levels of elderly colon cancer patients. Respecting the patients’ immersive humanistic needs for life interests, and exploring the adaptive leisure activities of classified nursing model from the perspective of interactive care should execute.

Adaptive leisure activities of the classified nursing model aim to combine an individual’s internal emotional factors, physiological status, and external situational factors to carry out appropriate leisure activities that enable individuals to pursue leisure enjoyment and participate in social interactions [5]. This model is designed to help the individual engage and get emotional counseling, spiritual pleasure, health guidance, and other purposes, as well as seek a positive subjective affective experience [6]. This nursing model focuses on the dual comfort experience of the individual’s physiology and psychology, and hopes to easily participate in a certain intervention activity from the perspective of humanistic immersion leisure activities, and obtain great happiness and satisfaction from it [7]. The model follows the principles of planning, systematization, and classification, examines the positive impact of leisure activities on the quality of life of individuals, and guides individuals to draw positive power from them, and formulates adaptive leisure activities based on factors such as individual physiological status, psychological quality, and external factors. This model can help individuals perceive the multidimensional positive factors of participating in leisure activities, stimulate individuals’ motivation and willingness to actively self-reflect, and continuously promote the rich interaction methods contained in in-depth leisure activities, thus having a significant impact on individuals’ positive emotional expectations [7]. During the implementation of adaptive leisure activities in the classified nursing model, individuals establish close interactive relationships with the surrounding people, help them integrate into organizational activities, build unique cultural and social attributes, create a happy and meaningful lifestyle, relieve negative emotions, and bring pleasant and positive efficacy.

Previous research may have focused more on the symptom management of colon cancer patients, while overlooking the psychological and emotional needs of these individuals [8]. This has resulted in limited emotional interaction between nurses and patients, leading to an ineffective means of comforting the negative emotions of colon cancer patients. Additionally, there may be a need to scientifically focus on the impact of interactive care on the adaptive leisure activities of these patients. This could involve studying the effectiveness of various leisure activities in promoting physical and mental well-being, as well as the ways in which interactive care can enhance the overall quality of life for elderly colon cancer patients. This study provides rehabilitation treatment of psychosocial function related to cases of different ages and genders, so that patients can feel the high-quality care model. This nursing model pays more attention to the construction of immersive leisure nursing activities, aiming to create a relatively warm and harmonious care atmosphere for patients, help patients obtain three-dimensional management at the level of psychological and social functions, and finally sublimate their psychological feelings. The rationale for this nursing program is based on the interactive construction between social norms and self-worth, effectively shaping the individual’s different expectations from the perspective of gender and age, so as to explore its impact on the individual’s psychosocial function and self-care behavior [9]. At present, this nursing model has not been fully promoted and popularized. This time, it is planned to further explore the nursing effect of its application in clinical practice through the design of relevant quantitative indicators with the help of randomized and controlled scientific research design schemes.

## Methods and materials

### Materials

In order to compare the effects of specific interventions or treatments, the research group and the control group selected different time periods. This design can help researchers assess the impact of interventions on outcomes while controlling for other potential influencing factors. Sample size calculation formula: *n* = 2σ2 (t α + t β)2/(μ 1-μ 2)^2^. Setting the two-sided test level α = 0.05, the test power β = 0.10, assuming a dropout rate of 20%, it can be seen that the minimum sample size for this study is 72 cases. Ultimately, 82 patients were selected. September 2017 to April 2020 and May 2020 to March 2022 were used as two study time periods from which colon cancer inpatients who met the inclusion and exclusion criteria were selected. In May 2020, the implementation of the Adaptive Leisure Activities of Classified Nurse Mode commenced. Consequently, employing May 2020 as the temporal demarcation point, we designated two comparable observation intervals: September 2017 to April 2020 and May 2020 to March 2022. The two patient groups both originate from the population of Cangnan County, China and sought medical treatment at Cangnan County People’s Hospital. By randomly selecting patient medical record numbers for those with computer implants, a lottery process was conducted to choose 41 cases for the research group and another 41 cases for the control group. The two groups of patients were named as the research group and the control group, with 41 patients in each group, setting inclusion conditions and exclusion criteria to select observation objects. Among them, the inclusion conditions were: ① The patient’s heart, liver, kidney and other important organs had good functions; ② The patients had good vision and hearing function, and was able to carry out basic interactive communication and dialogue; ③ The cognitive level of the patients was good; ④ Each patient shall be accompanied by at least one caregiver; ⑤ The patient was equipped with a smart phone and had the habit of using WeChat; ⑥ The patient was over 60 years old. Exclusion criteria: ① Patients with systemic immune system diseases; ② Patients with primary or secondary mental disorders; ③ The patient cannot complete the evaluation of the questionnaire or scale independently. The final included elderly patients with colon cancer were diagnosed as colon cancer by pathological biopsy. The diagnostic criteria were based on the guidelines of the national comprehensive cancer network. This study was approved by the hospital ethics committee.

## Methods

### Research group

The nursing model focuses on tailoring adaptive leisure activities for patients based on gender and age. This approach recognizes diverse needs, abilities, and preferences within different groups. For instance, older individuals may have distinct physical and cognitive abilities, while men and women may prefer different activities. Customizing activities for groups such as male and female patients with colon cancer, those aged 60–65, and those over 65 enhances engagement, satisfaction, and overall well-being. The goal is to address the unique requirements of each group through immersive, age- and gender-specific leisure programs.

### Male colon cancer patients

**Fig. 1:**
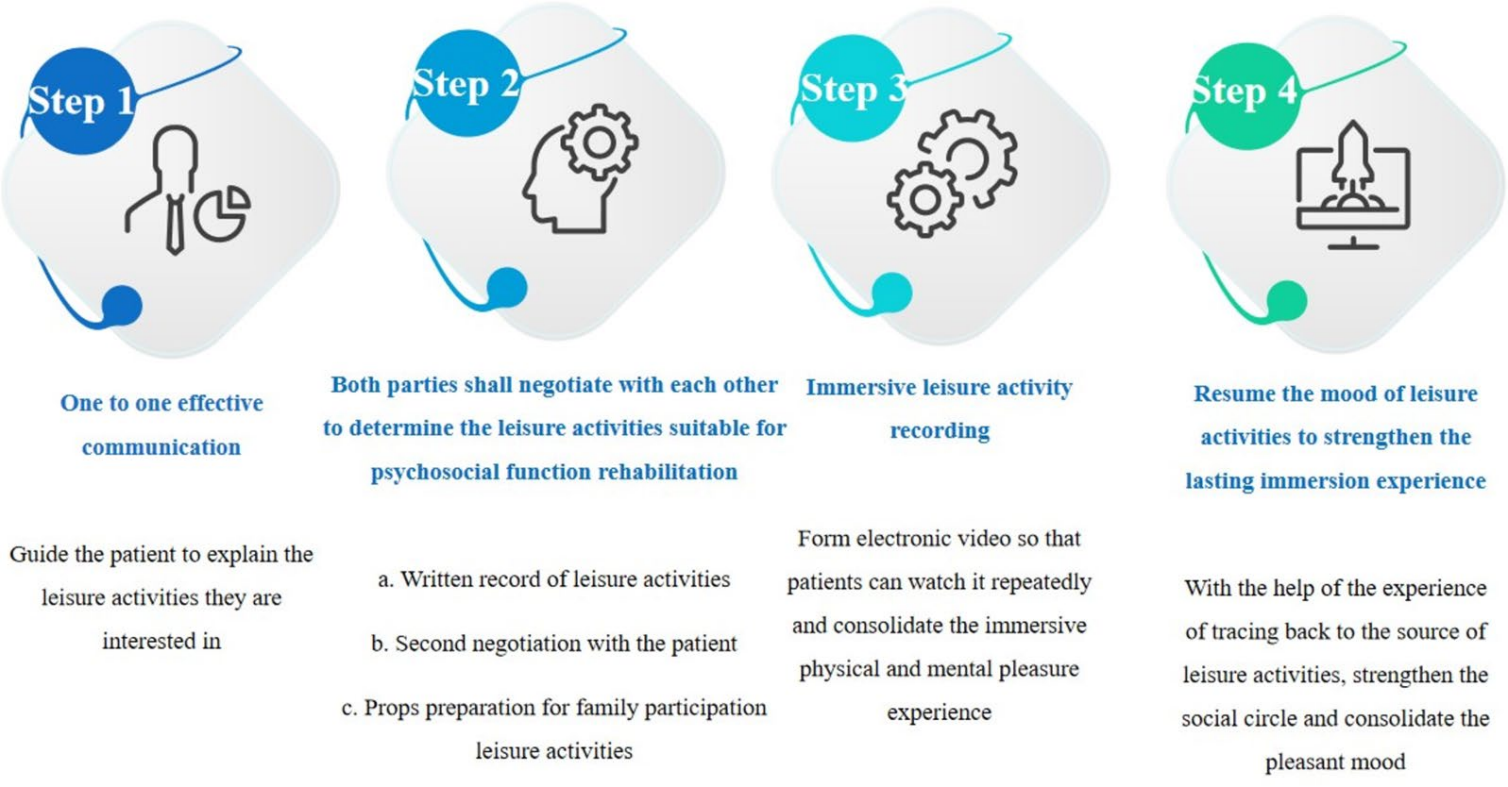
Nursing process of adaptive leisure activities for male colon cancer patients from the perspective of interactive care

Nurses address inner needs of male colon cancer patients through personalized leisure activities. The process involves those aspects. ① Private Space Creation: Nurses establish a private space for one-on-one communication. Patients share interests like calligraphy, chess, painting, and planting. ② Recording and Verification: Nurses record patient-described leisure activities. Verification and confirmation through communication with the patient. ③ Aerobic Exercise Consideration: Nurses factor in the patient’s aerobic exercise needs. Final activity selection based on hospital resources and patient consultation. ④ Preparation and Participation: Nurses and families prepare activity tools. Joint participation in leisure activities; recorded for later review. ⑤ Reflective Writing: Patients provided with pen and paper. Encouraged to write down and record their current state of mind. The nursing process enhances the well-being of male colon cancer patients through tailored, documented leisure activities (see Fig. 1).

### Female colon cancer patients

**Fig. 2:**
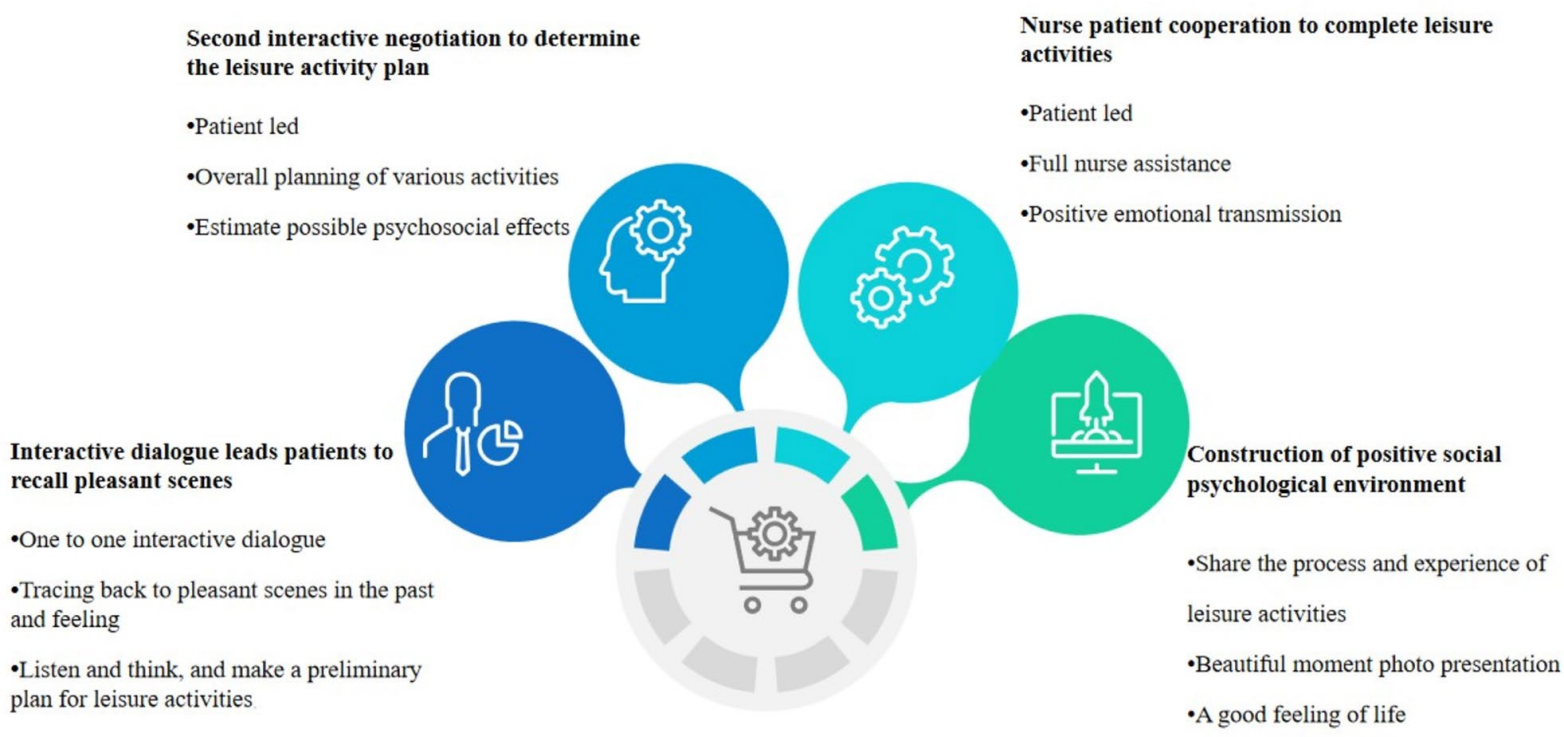
Adaptive leisure activity nursing process of female colon cancer patients from the perspective of interactive care

Nurses for female colon cancer patients adopt interactive care, understanding inner needs through one-to-one dialogue. The process involves those aspects. ① Emotion Recall and Identification: Nurses evoke past pleasant memories of leisure activities. Patients verbally describe memories, nurses listen, and interests are identified. ② Collaborative Planning: Secondary consultation for specific leisure plans. Patient-led formulation considering time, participants, tools, and psychological effects. ③ Coordinated Implementation: Nurses assist as patients lead planned activities. Verbal encouragement and behavioral support provided by nurses. ④ Sharing and Documentation: Patients host activities, sharing mental journeys and insights. Nurses document moments with photographs; patients express feelings on the back with a color pen. The nursing process, outlined in Fig. 2, involves collaborative planning and implementation, fostering psychological rehabilitation and well-being for female colon cancer patients.

### Patients aged 60∼65

**Fig. 3:**
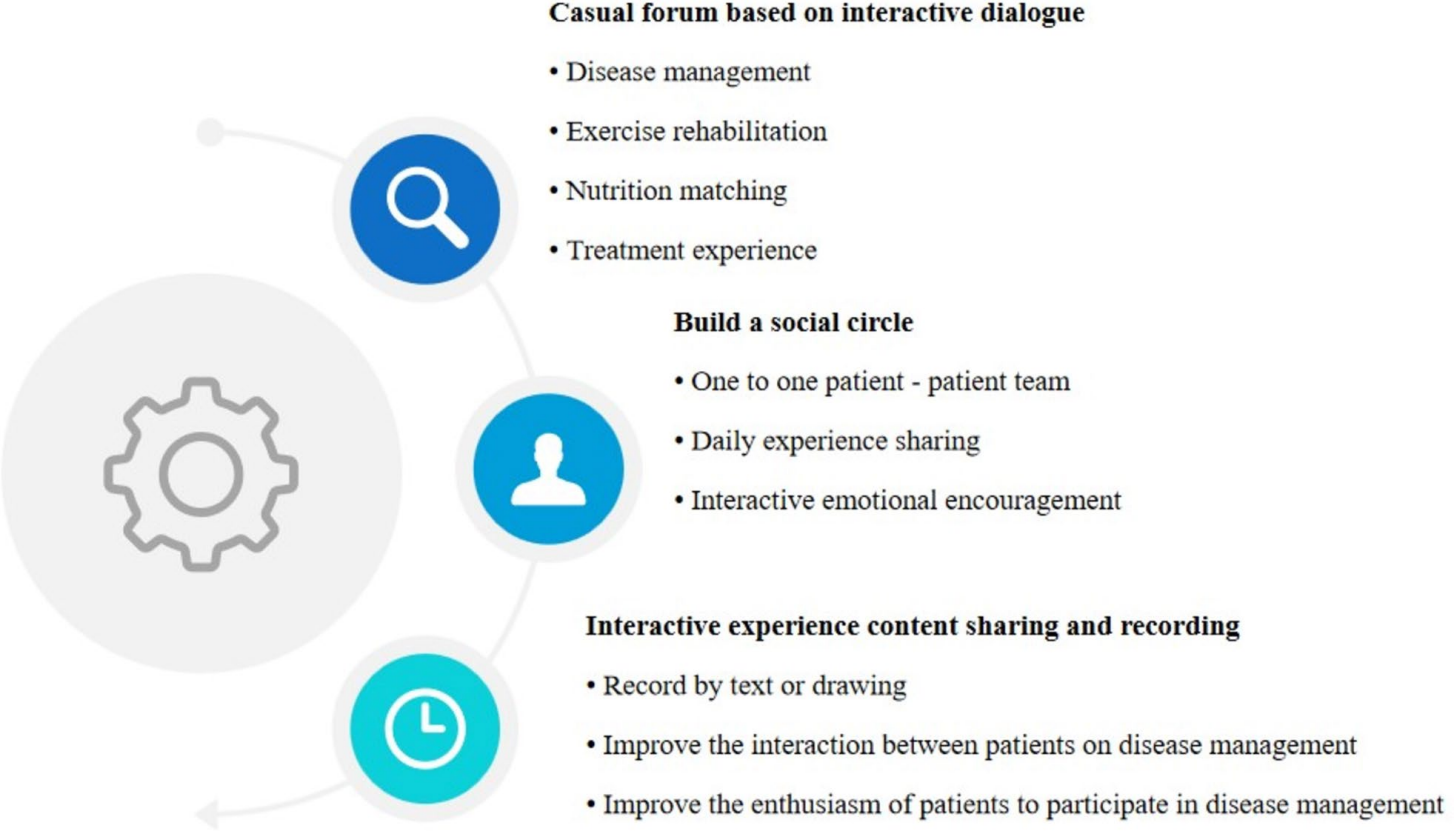
Nursing process of adaptive leisure activities for patients aged 60∼65 from the perspective of interactive care

Nurses of patients aged 60∼65 conducted nursing strategies of interactive leisure activities for colon cancer patients at this age. ① First of all, the symposium was held once a week by specially assigned persons, and the patients of this age group were invited to participate. As the moderator of the symposium, the nurses discussed the disease management, exercise rehabilitation, nutrition matching, treatment experience and other aspects that the elderly patients of this age group are interested in, so that each member can express their views and experiences. When each patient gave their own opinions, the nurse informed the patient in advance that they only needed to express the corresponding meaning clearly, and do not care about the logic of the speech. ② Secondly, the nurses encouraged the patients to form social circles through seminars and establish a one-to-one team, so that both parties could share their treatment experience every day and encourage each other. ③ Finally, the nurse distributed Notepad to each patient, told them to record the daily treatment experience in the form of text or drawings, and asked the patients in the team to supervise each other, so as to enhance the interaction and enthusiasm of each patient in participating in disease management. The nursing process of adaptive leisure activities for colon cancer patients aged 60∼65 is shown in Fig. 3.

### Patients over 65 years old

**Fig. 4:**
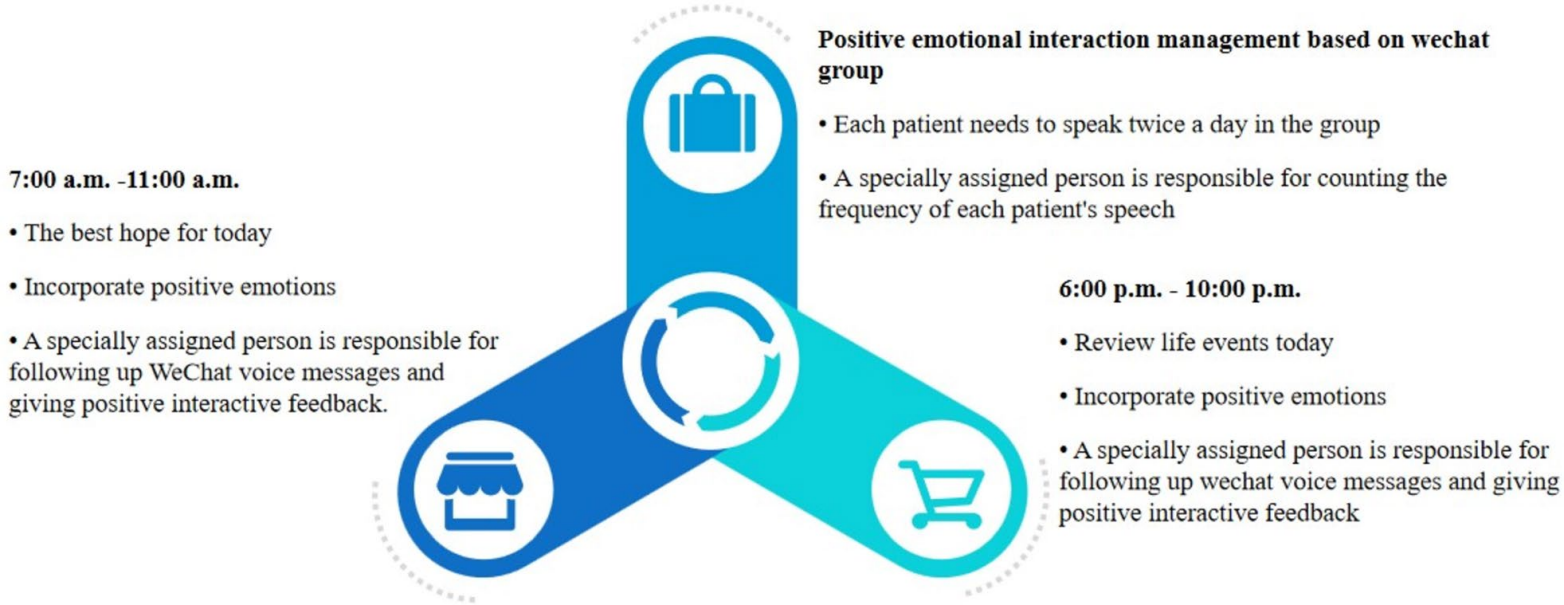
Nursing process of adaptive leisure activities for patients over 65 years old from the perspective of interactive care

① First, the nurse invited colon cancer patients over 65 years old to join the WeChat group, and informed each patient in advance about the rules related to speaking in the WeChat group, i.e. each patient had to speak twice a day in the group, and a special person was responsible for counting the frequency of each patient’s speaking. ② Second, from 7:00 a.m. to 11:00 a.m., the nurse invited the patients to send their best wishes for the day in the form of voice messages to the WeChat group. Once each patient left a message, a person was responsible for replying to the message from each patient. For those patients who have not sent voice messages at that time, a dedicated person would supervise them to complete the group message by telephone or on-site follow-up. ③ Moreover, between 6:00 p.m. and 10:00 p.m. the nurse invited the patients to review the day’s events, and send voice messages incorporating positive emotions. A dedicated person responded positively to the content of the patient’s voice message. For the patients who have not done in time, special personnel shall take the same method as above to supervise the patients to complete the group message. The nursing process of adaptive leisure activities for colon cancer patients over 65 years old is shown in Fig. 4.

### Control group

The traditional nursing care strategy was adopted for the patients in this group. The responsible nurse introduced the basic progress and prognosis of the disease to the patients and their families, provided basic life care to patients, guided them to change bed units, and kept the ward clean and dry, did a good job in basic drug care, and informed the efficacy of drugs and possible adverse events. In the aspect of symptom management, timely symptomatic treatment is a necessity for the nurse to ensure the stability of patient’s vital signs.

### Observation items

Both the research group and the control group patients were administered specific questionnaires or scales to assess their condition before and after the intervention.

### Self Rating Anxiety Scale (SAS) and Self Rating Depression Scale (SDS)

SAS and SDS [6] were prepared by Zung. Among them, mild anxiety has SAS score of 50 ∼ 59, moderate anxiety has SAS score of 60 ∼ 69, and severe anxiety has SAS score of more than 69. Mild depression has an SDS score of 53 ∼ 62, moderate depression has an SDS score of 63 ∼ 72, and severe depression has an SDS score higher than 72. Cronbach’s a of SAS scale was 0.823, kmo was 0.864, with good reliability and validity; Cronbach’s a of SDS was 0.851, kmo was 0.843, and the reliability and validity were good.

### Exercise of Self Care Agency (ESEA)

The ESEA scale [7] includes 4 dimensions and 43 items in total, including self-care skills (12 items), self-responsibility (8 items), self-concept (9 items) and health knowledge (14 items). Each item is scored by four levels (0–3 points). The higher the score of patients, the stronger the corresponding self-care skills. Cronbach’s a of the scale is 0.795 and kmo value is 0.827, with good reliability and validity.

### The MOS item short from health survey (SF-36)

The SF-36 scale [8] includes 8 dimensions: physical functioning, bodily pain, general health, role-physical, role-emotional, mental health, social functioning and vitality. The total score of the scale is 100. The higher the score, the better the corresponding quality of life of patients. Cronbach’s a of the scale is 0.843 and kmo value is 0.871, with good reliability and validity.

### Statistical methods

Spss22.0 software was employed for data entry, t-test was used for continuous data, and chi square test was adopted for discrete data. The difference was statistically significant (*p* < 0.05).

## Results

### Baseline data analysis

There was no significant difference in baseline data between the two groups (*p* > 0.05) (see Table 1).

**Table 1:** Comparison of general data between the two groups (*N* = 41)

Group		Research Group	Control group	Statistical value	*P*-value
**Male / Female**		22/19	23/18	0.049	0.824
**Age, years**		67.5 ± 4.2	67.7 ± 4.3	0.213	0.832
**Educational level**	**Primary school**	15	13	0.224	0.894
	**Middle school**	17	18		
	**University**	9	10		
**Tumor stage**	**Phase I**	8	10	0.286	0.867
	**Phase II**	19	18		
	**Phase III**	14	13		
**Body mass, kg**		65.9 ± 5.7	66.1 ± 5.8	0.157	0.875

### Comparison of affective traits between the two groups before and after intervention

Before intervention, there was no significant difference in anxiety and depression scores between the two groups (*P* > 0.05); After intervention, the scores of anxiety and depression in the research group were lower than those in the control group (*P* < 0.05) (see Table 2).

**Table 2:** Comparison of affective traits between the two groups before and after intervention

Group	Number of cases	Before intervention		After intervention	
		**Anxious**	**Depressed**	**Anxious**	**Depressed**
Research Group	41	61.5 ± 6.3	62.5 ± 7.1	52.9 ± 3.4	53.7 ± 5.1
Control group	41	61.7 ± 6.4	62.7 ± 7.2	59.1 ± 4.9	59.9 ± 6.4
*t*		0.143	0.127	6.656	4.851
*P*		0.887	0.900	< 0.001	< 0.001

### Comparison of ESEA Scale scores between the two groups before and after intervention

Before intervention, there was no significant difference in the total score and each dimension score of ESEA between the two groups (*P* > 0.05); After intervention,the self-concept, self-responsibility, self-care skills, health knowledge and total scores of the research group were significantly higher than those of the control group (*P* < 0.05) (see Table 3).

**Table 3:** Comparison of ESEA scale between the two groups before and after intervention(score) (*N* = 41)

Group	Before intervention					After intervention				
	**Self concept**	**Self responsibility**	**Self care skills**	**Health knowledge**	**Total score**	**Self concept**	**Self responsibility**	**Self care skills**	**Health knowledge**	**Total score**
Research Group	13.6 ± 2.3	12.8 ± 2.1	22.3 ± 6.1	40.9 ± 5.6	89.6 ± 7.6	18.4 ± 4.1	17.5 ± 3.6	28.5 ± 7.3	48.5 ± 7.0	112.9 ± 9.4
Control group	13.8 ± 2.4	12.6 ± 2.0	22.5 ± 6.2	41.1 ± 5.7	90.1 ± 7.8	14.6 ± 2.9	13.5 ± 2.2	23.4 ± 6.5	43.1 ± 6.2	94.6 ± 8.5
*t*	0.385	0.442	0.147	0.160	0.294	4.845	6.071	3.341	3.698	9.246
*P*	0.701	0.660	0.883	0.873	0.770	< 0.001	< 0.001	< 0.001	< 0.001	< 0.001

### Comparison of health status between the two groups before and after intervention

Before intervention, there was no significant difference in the total score and each dimension score of SF-36 between the two groups (*P* > 0.05); After intervention, the scores of physical functioning, bodily pain, general health, role-physical, role-emotional, mental health, social functioning and vitality in the research group were significantly higher than those in the control group (*P* < 0.05) (see Table 4).

**Table 4:** Comparison of quality of life scores between the two groups before and after intervention (score)

Observation items	Before intervention				After intervention			
	**Research Group(*N* = 41)**	**Control group (*N* = 41)**	***t***	***P***	**Research Group(*N* = 41)**	**Control group (*N* = 41)**	***t***	***P***
**Physical functioning**	63.9 ± 8.4	63.6 ± 8.2	0.164	0.870	86.2 ± 11.2	68.4 ± 8.4	8.141	< 0.001
**Bodily pain**	66.4 ± 9.0	66.7 ± 9.1	0.150	0.881	89.3 ± 14.6	72.7 ± 9.6	6.083	< 0.001
**General health**	53.8 ± 6.1	54.1 ± 6.2	0.221	0.826	89.4 ± 14.1	66.1 ± 7.2	9.424	< 0.001
**Role-Physical**	62.8 ± 5.7	62.5 ± 5.6	0.240	0.811	86.4 ± 9.6	71.3 ± 7.2	8.057	< 0.001
**Role-Emotional**	52.9 ± 4.4	53.1 ± 4.5	0.203	0.839	83.5 ± 8.6	62.7 ± 5.2	13.252	< 0.001
**Mental health**	53.8 ± 5.7	54.1 ± 5.8	0.236	0.814	79.5 ± 7.7	60.1 ± 6.2	12.565	< 0.001
**Social functioning**	44.5 ± 4.9	44.7 ± 5.0	0.183	0.855	73.4 ± 11.0	52.4 ± 5.8	10.813	< 0.001
**Vitality**	55.2 ± 5.9	55.6 ± 6.0	0.304	0.762	79.1 ± 8.3	57.5 ± 6.8	12.890	< 0.001

## Discussion

### Essentiality of adaptive leisure for elderly colon cancer patients

Elderly colon cancer patients are suffering from negative emotions as a result of the long-term disease and the toxic side effects of the treatment regimen [10]. How to excavate the humanistic appeal of individuals from the perspective of life interest and guide individuals to face the disease with an optimistic and positive attitude will promote the rehabilitation of the psychosocial function of cancer patients, make them calmly deal with the disease and improve their quality of life [11]. The traditional nursing intervention regards the patient as a passive individual, and implements corresponding management only according to the physical symptoms, thus ignoring the internal demands of the patient at the level of mental psychology and cognitive function, which is not conducive to the patient’s emotional management and quality of life improvement [12]. In this study, we put forward the adaptive classified leisure nursing model based on the perspective of life interest, which is a specific manifestation of respecting the humanistic demands of cancer patients, through the comprehensive management of the mental, psychological and social functions of individual patients [13]. Enjoying adaptive leisure activities is a more objective and real life style of individuals, and different ages and genders will also lead to specific perceptual experiences of psychosocial functions [14]. Therefore, guided by the most real humanistic demands of individuals, we should explore immersive leisure activities based on life interests to help patients obtain stable emotional perception during the treatment process and enhance their social circle, so as to promote the rehabilitation of psychological and social functions of colon cancer patients.

### Impact of adaptive leisure on elderly colon cancer patients

In this study, the SAS and SDS scores of the research group after intervention were lower than those of the control group, and the difference was statistically significant (*p* < 0.05), indicating that adaptive immersive leisure activities from the perspective of interactive care can help to improve the negative emotional status of patients. This is mainly because nurses always respect the immersive humanistic demands of elderly colon cancer patients on life interests during the whole care process, and try to meet the patients’ different expectations on leisure activities in terms of gender and age. However, there is a close relationship between leisure activities and age and gender. Therefore, when implementing the design of interactive leisure activities, it is necessary to comprehensively evaluate the gender and age of patients and provide them with appropriate leisure activities [15]. Among them, compared with the elderly patients over 65 years old, the elderly patients aged 60 to 65 years old are more likely to be guided by their hobbies in leisure activities. If their humanistic demands for life interests are respected, they can mobilize their internal positive emotions to stimulate them to participate in social activities, so as to improve their relatively negative psychological conditions. When the patients’ life interests are basically satisfied, they can ensure that their body and mind are in a relaxed state, so that they can no longer stick to the past or the future, and take the initiative to face every day of life [16]. Adaptive leisure activities can help patients actively pursue a better quality of life, make them feel free and comfortable, and make them produce “extremely happy” psychosocial behavior [17]. Carry out classified leisure nursing experience mode for patients of different ages and genders, which can provide them with real happiness perception experience, make patients aware of the importance of a sense of freedom, and thus vent their internal negative perception emotions [18]. After the patients experienced the adaptive classified leisure activities throughout, with the help of text, audio, drawings and other measures, they can reshape their self-perception, form positive cognition, and thus experience the life journey in a realistic way [19].

### Effects of adaptive immersion leisure activities on self-care behavior and health status of elderly patients with colon cancer from the perspective of interactive care

By comparing the self-care behavior of the two groups, we found that the self-care behavior of the research group was better than that of the control group (*p* < 0.05). This shows that adaptive immersion leisure activities can improve patients’ self-efficacy from the perspective of interactive care. As people grow older, they will surely focus on a group of partners with the same interests and similar environments. These individuals can participate in a certain activity together to realize the connection between life and spiritual emotion. Therefore, by setting up symposiums and WeChat groups, the social ways are diversified, so that the elderly colon cancer patients of different ages and genders can have a more satisfactory knowledge and practice experience [16]. In this process, patients can improve their daily living conditions, so as to give self-understanding and self-acceptance, which can produce continuous self-recognition, thus improving individual self-care behavior [20]. In addition, with the growth of age, patients need to face a variety of different problems, that is, on the basis of different gender and age, they have different lifestyles [21]. With the gradual implementation of adaptive immersion leisure activities from the perspective of interactive care, it can help elderly colon cancer patients to improve their psychosocial functions in the process of communication and self-expression. Moreover, the nurse patient and patient patient interaction process, in fact, is to promote the effective expression of patients’ psychology and emotion, and it is another level of leisure activities [22]. There is evidence that individuals can achieve satisfaction in their favorite leisure activities and are motivated to engage in leisure activities on an ongoing basis, which can effectively maintain their physical and mental health [23]. As a result, patients in the research group have experienced significant improvements in psychosocial and self-efficacy after receiving the adaptive leisure nursing model from the gender and age perspective, which contributed to their improved health status [24].

### Limitations and future research prospects

#### Limitations

The limitations of this study may include potential sample bias, as the life interests of elderly colon cancer patients are influenced by individual differences, which may limit the generalizability of the research findings. Additionally, subjective biases in the study could be a limitation, as the personal views and experiences of the researchers may impact the exploration of immersive humanistic appeals and the adaptability of leisure activities and nursing classification models.

#### Future research prospects

Future research prospects include expanding the sample size to increase the reliability and generalizability of the research findings. Additionally, utilizing multiple research methods, such as combining qualitative and quantitative approaches, to gain a more comprehensive understanding of the immersive humanistic appeals and the needs for interactive care and leisure activities among elderly colon cancer patients. Furthermore, future research could delve into investigating the impact of different types of leisure activities on the physical and mental health of elderly colon cancer patients, as well as evaluating the effectiveness of different nursing classification models in practical application. Lastly, considering conducting similar studies among other chronic disease patient groups to compare their needs for interactive care and leisure activities and their adaptability.

## Conclusion

In summary, the application of Adaptive Immersion Leisure Activities, integrated with a Classified Nursing Model and an Interactive Care approach, effectively contributed to a significant reduction in psychological distress and enhancement of various aspects of well-being among elderly colon cancer patients. These findings underscore the importance of personalized, interactive interventions in promoting a holistic improvement in the health and quality of life for this patient population.

## Clinical implications

From the perspective of interactive care, exploring the adaptive leisure activities of classified nursing mode can meet the immersive humanistic demands of elderly colon cancer patients for life interests, strengthen individual psychosocial functions, enable them to actively participate in disease management, reduce negative emotions, obtain inner and outer well-being, and ultimately improve an individual’s quality of life.

## Data Availability

The original contributions presented in the study are included in the article, further inquiries can be directed to the corresponding author/s.
