# Correction: Exploring the adaptive leisure activities of classified nursing model in elderly colon cancer patients: a perspective on interactive care

**DOI:** 10.1186/s12904-023-01337-8

**Published:** 2024-01-05

**Authors:** Yun‑Zhao Lin, Po Chen, Meng‑Meng Lin, Jia‑Li Chen, Min‑Min Shi, Feng Guo

**Affiliations:** 1 Hangzhou City University, Hangzhou, China; 2 Saint Louis University, Baguio, Philippines; 3 Zhejiang Wanli University, Ningbo, China; 4 Ningbo Wanli Counterpart Cooperation and Anti-Poverty Research Institute, Ningbo, China; 5 Cangnan County People’s Hospital, Wenzhou, China; 6 School of Nursing, Zhejiang Chinese Medical University, Hangzhou, China; 7 School of Management, Wenzhou Business College, Wenzhou, China

**Correction: BMC Palliat Care 22, 198 (2023)**

10.1186/s12904-023-01317-y

Following publication of the original article [1], the author reported that the first authors’primary affiliation should only be the one to be captured. Moreover, Yun-Zhao Lin should be treated as a joint corresponding author. Remove co-first authorship for Jia-Li Chen.
